# Influence of Obstructive Sleep Apnea on Systemic Inflammation in Pregnancy

**DOI:** 10.3389/fmed.2021.674997

**Published:** 2021-11-02

**Authors:** Alberto Alonso-Fernández, Caterina Ribot Quetglas, Andrea Herranz Mochales, Ainhoa Álvarez Ruiz De Larrinaga, Andrés Sánchez Barón, Paula Rodríguez Rodríguez, Ana Victoria Gil Gómez, Carla Pía Martínez, José Pablo Cubero Marín, Maria Barceló Nicolau, María Cerdà Moncadas, Mercedes Codina Marcet, Mónica De La Peña Bravo, Antònia Barceló Bennasar, Amanda Iglesias Coma, Daniel Morell-Garcia, José Antonio Peña Zarza, María Paloma Giménez Carrero, Joaquín Durán Cantolla, José María Marín Trigo, María Concepción Piñas Cebrian, Joan B. Soriano, Francisco García-Río

**Affiliations:** ^1^Institut d'Investigació Sanitària Illes Balears (IdISBa), Palma, Spain; ^2^Servicio de Neumología, Hospital Universitari Son Espases, Palma, Spain; ^3^Centro de Investigación Biomédica en Red de Enfermedades Respiratorias (CIBERES), Madrid, Spain; ^4^Unidad del Sueño, Hospital Universitario de Araba, Vitoria-Gasteiz, Spain; ^5^Instituto de Investigación BIOARABA, Vitoria-Gasteiz, Spain; ^6^Servicio de Neumología, Hospital Universitario Miguel Servet, Zaragoza, Spain; ^7^Instituto de Investigación Sanitaria de Aragón, Hospital Universitario Miguel Servet, Zaragoza, Spain; ^8^Servicio de Neumología, Hospital Palma Planas, Palma, Spain; ^9^Servicio de Endocrinología, Hospital Universitari Son Espases, Palma, Spain; ^10^Servicio de Análisis Clínicos, Hospital Universitari Son Espases, Palma, Spain; ^11^Servicio de Pediatría, Hospital Universitari Son Espases, Palma, Spain; ^12^Hospital Universitario La Princesa, Universidad Autónoma de Madrid, Madrid, Spain; ^13^Servicio de Neumología, Hospital Universitario La Paz, Instituto de Investigación del Hospital Universitario La Paz (IdiPAZ), Madrid, Spain

**Keywords:** apneas-hypopneas index, hypoxia, fetal outcomes, REM, cytokine, inflammation, Obstructive sleep apnea

## Abstract

**Background:** Obstructive sleep apnea (OSA) is prevalent in pregnancy and it is associated with adverse pregnancy-related outcomes such as gestational diabetes, pre-eclampsia, and low birth weight. Maternal systemic inflammation is proposed to be one of the main intermediate mechanisms. However, the effects of OSA on systemic inflammation are unknown in normal pregnancy.

**Methods:** Women in the 3rd trimester underwent hospital polysomnography to evaluate whether OSA increases systemic inflammation in normal pregnancy and its potential association with adverse fetal outcomes. OSA was defined as an apnea–hypopnea index (AHI) of ≥ 5 h^−1^. Plasma cytokines levels (TNF-α, IL-1β, IL-6, IL-8, and IL-10) were determined by multiple immunoassays.

**Results:** We included 11 patients with OSA and 22 women with AHI < 5 h^−1^, who were homogeneous in age, and body mass index (BMI). Women with OSA had significant higher levels of TNF-α, IL-1β, IL-8, and IL-10. We found significant correlations between AHI during REM and TNF-α (*r* = 0.40), IL-1β (*r* = 0.36), IL-6 (*r* = 0.52), IL-8 (*r* = 0.43), between obstructive apnea index and TNF-α (*r* = 0.46) and between AHI and IL-1β (*r* = 0.43). We also found that CT90% was related to IL-8 (*r* = 0.37). There were no significant differences in neonatal characteristics; however, we found inverse correlations between TNF-α and IL-8 with birth weight (both *r* = −0.48), while IL-8 showed a significant inverse relationship with neonatal gestational age (*r* = −0.48).

**Conclusions:** OSA in our normal pregnancy population was associated with higher systemic inflammation, which was related to obstructive events, especially during REM sleep. Moreover, systemic inflammation was inversely correlated with neonatal birth weight and age.

## Introduction

Obstructive sleep apnea (OSA) is a common disorder characterized by the presence of repetitive episodes of total (apneas) or partial (hypopneas) airflow cessation in the upper airway during sleep despite increased respiratory effort (1). Apneas and hypopneas are frequently accompanied by a decrease in arterial oxygen saturation that normalizes with breathing resumption producing repeated cycles of hypoxia-reoxygenation (2), which are associated to an increase in pro-inflammatory cytokines in general population (3, 4). These cytokines are secreted in different tissues, including adipose tissue in a close relationship between obesity and OSA. Obesity is the primary risk factor for OSA. In fact, other studies have already shown the relationship between weight gain and the risk of OSA or increasing its severity (5).

Prevalence of OSA during pregnancy increases due to some physiological changes, such as weight gain or modifications in the upper airway (6, 7). OSA is associated with adverse pregnancy outcomes such as gestational diabetes mellitus (GDM) (8–10), pre-eclampsia (8), gestational hypertension (11), and with fetal related outcomes (12, 13), preterm birth, and neonatal low weight (14). Normal pregnancy is associated with higher inflammation markers compared to non-pregnant women (15–17). Patients with GDM or pre-eclampsia show further increments in inflammation levels (18–21). There are also significant associations between maternal systemic inflammation and neonatal low birth weight (20, 22) even though more studies are clearly required to clarify the influence of important confounding factors such as obesity, age and comorbidities. It has been proposed that increments in systemic inflammation (23) are one of the main mechanisms that associate OSA with adverse pregnancy outcomes. However, no studies have assessed the effect of OSA on systemic inflammation in uncomplicated pregnancies so far.

The study aims were: (1) to evaluate the levels of inflammatory cytokines (tumor necrosis factor alpha (TNF-α), interleukin 1 beta (IL-1β), interleukin 6 (IL-6), interleukin 8 (IL-8), and interleukin 10 (IL-10) in OSA and healthy pregnant women who were homogeneous in age, and body mass index (BMI) in the third trimester; (2) to relate systemic inflammation with adverse maternal and fetal outcomes.

## Materials and Methods

### Subjects and Study Design

Women were recruited at three tertiary and university hospitals in Spain (Hospital Universitari Son Espases, Hospital Universitario Araba and Hospital Universitario Miguel Servet). Inclusion criteria were: (1) singleton pregnant women in the third trimester of pregnancy, and (2) signed an informed consent (IC). Patients were excluded if they fulfilled at least one of the following exclusion criteria: (1) previous OSA diagnosis, (2) glucose value at 1 h after a 50-g oral glucose administration that did exceed 140 mg/dL, (3) complicated pregnancy (gestational hypertension, GDM, pre-eclampsia, and any other significant obstetrical complication), (4) previous diabetes mellitus, pulmonary, cardiac, or kidney diseases, (5) treatment with steroids, (6) unwillingness or inability to participate in the study, (7) imminent delivery due to maternal-fetal disease; and/or (8) any other concurrent severe medical condition that would, in the investigators' judgment, contraindicate patient participation in the study. Women were selected from a large study evaluating consequences of OSA during pregnancy. Based on previous studies reporting plasma TNF-α levels of 4.6 ± 0.6 pg/mL in non-complicated third trimester of pregnancy (24) with an alpha risk of 0.05 and a beta risk in a two-way contrast, 22 healthy pregnant women and 11 pregnant women with OSA are required to detect a difference equal to or >0.63 pg/mL (14% of mean value).

### Ethics Statement

The study was approved by the Institutional Ethics Committee of the hospitals and all subjects gave their written informed consent.

Each patient had their anthropometric, clinical, and sleep data collected by means of questionnaires and direct measurements that included: age, body mass index, co-morbidities and alcohol intake and tobacco consumption. Neck, waist, and hip circumferences were measured at the levels of the cricothyroid membrane, at the point equidistant between the iliac crest and the lowest rib, and at the point of greater trochanter, respectively. Office blood pressure was measured by a random-zero sphygmomanometer with appropriate sized cuff, with the individual seated for at least 5 min. Recorded values were the mean of three readings.

Episodes of subjective gasping, snoring, bed partner-reported sleep breathing pauses, nocturia, morning headache, morning tiredness, and sleepiness while driving were collected in four degrees of intensity (never, sometimes, frequently, and always) based on the previous 4 weeks. Subjective nocturnal sleep time as well as napping time on working days and weekends along with daytime sleepiness [using the Epworth sleepiness scale (ESS) (25)] based on the previous 4 weeks were ascertained. Daytime sleepiness was defined as an ESS ≥ 11.

### Polysomnography (PSG)

Overnight-attended PSG was performed in the sleep laboratory. Electroencephalogram (C3-A2, C4-A1), electrooculogram, chin electromyogram, electromyograms of the tibialis anterior of both legs and electrocardiogram were continuously recorded. Breathing was monitored using nasal cannulas, oronasal thermistors, and thoracoabdominal strain gauges. Simultaneously, oxyhemoglobin saturation (SpO_2_) was monitored with a pulse oximeter. Sleep was analyzed using the standard criteria for epochs of 30 s (26). Apnea was defined as the absence of airflow (>90% reduction) for at least 10 s while hypopnea was defined as a discernible airflow reduction (>30% and <90%) for at least 10 s with a ≥3% drop in oxygen saturation or final arousal. The events were considered obstructive in the presence of continued respiratory efforts. The apneas-hypopneas index (AHI) was established as the number of apneas and hypopneas per hour of sleep. OSA was defined as an AHI of ≥ 5 h^−1^ (27). REM AHI was calculated as the number of apneas and hypopneas during REM sleep divided by total time in REM sleep in hours. As indices of nocturnal oxygen saturation, the medium SpO_2_ throughout the night, the minimum SpO_2_ (lowest values recorded during sleep) and total time study spent with SpO_2_ <90% (CT90%) were computed.

### Blood Sample Collection and Determinations

The morning after the PSG, anthropometric variables, and blood samples were collected from all women in fasting conditions. Laboratory data included complete blood count (Cell-Dyn Sapphire Platform, Abbott Diagnostics), coagulation, kidney and liver function tests (Architect c16000 platform. Abbott Diagnostics, US). Insulin was determined by processing serum samples on the Cobas e-411 platform (Roche Diagnostics GmbH, Germany) with a reference range of 3–25 μUI/mL. The Homeostatic model assessment of insulin resistance index (HOMA-IR) was calculated by the usual formula (28).

The remaining blood sample was centrifuged at 2,500 rpm for 10 min to isolate the plasma. The different plasma aliquots were stored at −80**°**C for further determinations.

The inflammatory cytokines were analyzed on plasma samples by multiplex technique using the Human High Sensitivity Cytokine Base Kit A (Magnetic Luminex® Performance Assay, R&D Systems®, Inc.) following the indicated procedure. For each one of the cytokines, analyzed the detection limits and coefficients of variation were 0.29 pg/mL and 5.2% (TNF-α), 0.08 pg/mL and 5.3% (IL-1β), 0.14 pg/mL and 5.2% (IL-6), 0.04 pg/mL and 6.6% (IL-8), 0.21 pg/mL and 5.4% (IL-10).

Following the delivery day, anthropometric and neonatal variables were collected from all newborns. Laboratory researchers were blinded to maternal status.

### Statistical Analysis

Data are presented as mean ± standard deviation, median + interquartile range (IQR) or percentage. Differences between groups were analyzed using Student's *t*-test or *U* Mann-Whitney test for continuous variables, and Fisher's exact test (two-tailed) or chi-squared test for categorical variables. As total sleep time (TST) was significantly different between apneic and non-apneic pregnant women, plasma levels of inflammatory biomarkers were corrected using a General Linear Model univariate procedure with group (OSA vs. non-OSA) as a fixed-effect factor and TST as a covariate factor. Moreover, inflammatory biomarkers were corrected using a General Linear Model univariate procedure with group (OSA vs. non-OSA) as a fixed-effect factor, and BMI before pregnancy and gestational age at plasma sampling, and BMI and gestational age at plasma sampling as covariate factors, respectively. Pearson's and Spearman's correlation tests examined correlations between variables. A two-sided *P* < 0.05 was considered significant.

## Results

### Subjects Study

To obtain the sample size pre-determined for the experimental group, and to include 11 pregnant women classified as OSA, we required assessing for eligibility 130 pregnant women, 42 were not selected (25 refused, 1 technical sleep study drop-out, 1 twin pregnancy, 4 delivery before PSG, 1 change of address), and we finally needed to perform PSG to 88 women. We selected 22 women among the remaining non-OSA group, who were homogeneous in age, and body mass index (BMI) for comparative purposes. All 33 pregnant women were Caucasian except for one Hispanic. The descriptive analysis of both groups for their main anthropometric and clinical variables as well as the laboratory findings are shown in Table 1. The anthropometric characteristics, smoking status, nulliparous percentages, number of previous pregnancies, and blood pressure did not differ between patients with OSA and controls. Plasmatic fasting glucose levels were higher in non-OSA women than in the OSA group.

**Table 1:** General characteristics of pregnancy women with and without OSA.

**Variables**		**Total**	**OSA** **(***n*** = 11)**	**Non-OSA** **(***n*** = 22)**	***P*** **-value**
Age (years)		35 ± 3.9	33.9 ± 5	35.6 ± 3.1	0.245^¥^
Gestational age (weeks) at plasma sampling		33.5 ± 3	34.5 ± 3	33.1 ± 3	0.230^¥^
BMI before pregnancy (Kg/m^2^)		22.6 ± 3.4	23 ± 3.4	22.4 ± 3.4	0.641^¥^
Obese before pregnancy *n* (%)		2 (6.1)	0	2 (9.1)	0.542^∞^
BMI at plasma sampling (Kg/m^2^)		26.8 ± 2.9	27.2 ± 2.4	26.6 ± 3.2	0.555^¥^
First pregnancy *n* (%)		17 (51.5)	6 (54.5)	11 (50)	0.805^∞^
Pre-gestational smoker *n* (%)	No	21 (63.6)	7 (63.6)	14 (63.6)	0.710^∞^
	Yes	7 (21.2)	3 (27.3)	4 (18.2)	
	Former smoker	5 (15.2)	1 (9.1)	4 (18.2)	
Pre-gestational tobacco dose (packs/year)		2 (0–10)	0 (0–10)	2.75 (0–10)	0.428^¶^
Gestational smoker *n* (%)		4 (12.1)	1 (9.1)	3 (13.6)	0.427^∞^
Neck circumference (cm)		33.5 ± 2.3	34 ± 1.7	33.3 ± 2.6	0.403^¥^
Waist (cm)		100.7 ± 12	103.9 ± 5.8	99 ± 14	0.284^¥^
Hip circumference (cm)		104.5 ± 6.9	103.7 ± 6.2	104.9 ± 7.6	0.767^¥^
Glucose (mg/dL)		73.3 ± 8.5	69.2 ± 7.9	75.1 ± 8.3	0.068^¥^
Insulin (μUI/mL)		10.3 (7.6–14.5)	13.3 (9.7–15)	9.4 (7.5–12.9)	0.108^¶^
HOMA-IR		1.8 (1.3–2.6)	2.6 (1.3–3)	1.6 (1.3–2.4)	0.327^¶^
Total cholesterol (mg/dL)		279 ± 49	286 ± 57	274 ± 46	0.537^¥^
Triglycerides (mg/dL)		189 (163–237)	221 (179–279)	179 (160–219)	0.122^¶^
HDL (mg/dL)		72 ± 13	68 ± 15	73 ± 12	0.311^¥^
LDL (mg/dL)		16 ± 3	173 ± 43	166 ± 433	0.651^¥^
ALT (U/L)		13 (10–17)	16 (10–28)	13 (10–16)	0.338^¶^
GGT (U/L)		9 (7–13)	9 (8–17)	9 (7–12)	0.644^¶^
Hemoglobin (g/dL)		11.9 ± 0.9	11.4 ± 0.9	12.1 ± 0.9	0.084^¥^
Leucocytes (10^3^/uL)		9.75 ± 2.16	10.52 ± 2.25	9.4 ± 2.08	0.178^¥^
Platelets (10^3^/uL)		226.81 ± 52.39	246.8 ± 41.68	217.7 ± 55.05	0.149^¥^
Systolic BP (mmHg)		105 ± 9	105 ± 11	105 ± 9	0.990^¥^
Diastolic BP (mmHg)		65 ± 7	67 ± 9	64 ± 6	0.263^¥^

*Descriptive analysis and comparison between groups. Continuous variables in mean ± SD if they follow normal distribution or in median (IQR) if they follow a non-parametric distribution. For categorical variables n (%). Statistical tests, t-Student^¥^; U Mann- Whitney^¶^; Chi-square^∞^. BMI, body mass index; HOMA-IR, homeostasis model assessment of insulin resistance; HDL, high-density lipoprotein; LDL, low-density lipoprotein; ALT, alanine aminotransferase; GGT, gamma glutamyl-transpeptidase; BP, blood pressure; g, grams; Kg, kilograms; mg, milligrams; m, meter; cm, centimeter; dL, deciliter; U, units; L, liter, uL, microliter; mmHg, millimeter of mercury*.

We included 11 pregnant women with OSA (AHI median 7.8 (IQR 5.6–9.7) h^−1^) and 22 non-OSA (AHI median 0.35 (IQR 0.07–0.7) h^−1^). Table 2 illustrates differences in PSG characteristics, symptoms and anthropometrics between groups. TST was longer in non-OSA women than in OSA subjects. As expected, there were significant differences between groups in the AHI, REM AHI, the obstructive apneas index (OAI), the desaturations index, and minimum SpO_2_ (Table 2). Three women had ESS ≥ 11, and all of which presented AHI < 5 h^−1^. No differences were found in daytime somnolence, snoring, episodes of subjective asphyxia, nocturia, morning headache, reported apneas, unrefreshing sleep, and reported sleep time between OSA and controls (Table 2). In addition, we did not find differences in specific OSA physical examination.

**Table 2:** Polysomnography data, sleep symptoms, and physical characteristics in OSA and non-OSA groups.

**Variables**		**Total**	**OSA** **(***n*** = 11)**	**Non-OSA** **(***n*** = 22)**	***P*** **-value**
Total sleep time, (min)		306 ± 66	269 ± 74	325 ± 55	**0.02^¥^**
N1 + N2 sleep time (%)		64.1 (59.8–72.5)	60.9 (20.1–66.1)	66.2 (60.7–73.1)	0.127^¶^
N3 sleep time (%)		22.1 (16.8–27.1)	26.4 (16.2–57.2)	19.7 (16.9–23.6)	0.127^¶^
REM sleep time (%)		11.69 ± 5.66	11.43 ± 7.55	11.83 ± 4.64	0.852^¥^
AHI (h^−1^)		0.7 (0.2–6)	7.8 (5.6–9.7)	0.35 (0.07–0.7)	**0.001^¶^**
OAI (h^−1^)		0 (0–0.1)	0.2 (0–0.4)	0 (0–0)	**0.004^¶^**
Arousal index (h^−1^)		9.9 (0.25–19.6)	16.10 (6.8–19.9)	6.10 (0–17.98)	0.098^¶^
REM AHI (h^−1^)		1 (0–5.65)	8.4 (1.7–23.5)	0 (0–1.48)	**0.001^¶^**
Mean SpO_2_ (%)		96 (95–97)	96 (95–97)	96 (95–97)	0.546^¶^
Minimum SpO_2_ (%)		92 ± 2	91 ± 3	93 ± 2	**0.013^¥^**
CT90%SpO_2_ (min)		0 (0–0)	0 (0–0.1)	0 (0–0)	0.08^¶^
Desaturation index (h^−1^)		0.1 (0–0.6)	1.10 (0.3–2.3)	0 (0–0.15)	**0.001^¶^**
Nocturia *n* (%)		29 (87.9)	9 (81.8)	20 (90.9)	0.407^∞^
Reported apneas, *n* (%)		2 (6.1)	2 (18.2)	0 (0)	0.104^∞^
Frequent gasping awakenings, *n* (%)		4 (12.1)	3 (27.3)	1 (4.5)	0.097^∞^
Morning headaches *n* (%)		4 (12.1)	3 (27.3)	1 (4.5)	0.097^∞^
Unrefreshing sleep *n* (%)		20 (60.6)	7 (63.6)	13 (59.1)	0.801^∞^
Snoring (always/frequent) *n* (%)		8 (25)	3 (27.3)	5 (23.8)	0.830^∞^
Reported sleep time, working days (h)		7 (6–8)	6 (4–8)	7 (6–8)	0.402^¶^
Reported sleep time, weekends (h)		7.5 (6–8.3)	6 (4–8.5)	7.5 (7–8)	0.576^¶^
Micrognatia n (%)		2 (6.1)	0 (0)	2 (9)	0.542^∞^
Mallampati score n (%)	I–II	26 (81.3)	8 (72.7)	18 (85.7)	0.390^∞^
	III–IV	6 (18.8)	3 (27.3)	3 (14.3)	
Epworth sleepiness scale		6.5 ± 3	6.7 ± 2.1	6.4 ± 3.4	0.776^¥^

*Continuous variables in mean ± SD if they follow normal distribution or in median (IQR) if they follow a non-parametric distribution. For categorical variables n (%). Statistical tests: t-Student^¥^; U Mann- Whitney^¶^; Chi-square^∞^. N1+N2, superficial sleep time; N3, deep sleep time; REM, rapid eye movement; AHI, apneas/hypopneas index; OAI, obstructive apneas index; SpO_2_, oxygen saturation; CT90%, sleep time with oxygen saturation <90%; min, minutes; h^−1^, per hour; h, hours. Values in bold indicate significant difference*.

Cesarean section was performed in 18%, 2 (18.2%) in OSA group, and 4 (18.2%) in non-OSA pregnant women. The deliveries were instrumented in 21, and 24% were induced deliveries; there were no miscarriages. None of them had vaginal candidiasis, pre-eclampsia, or proteinuria, but 7.1% had urinary infection. There were no significant differences between OSA and non-OSA pregnant women in all these clinical data.

### Inflammatory Profile

**Figure 1:**
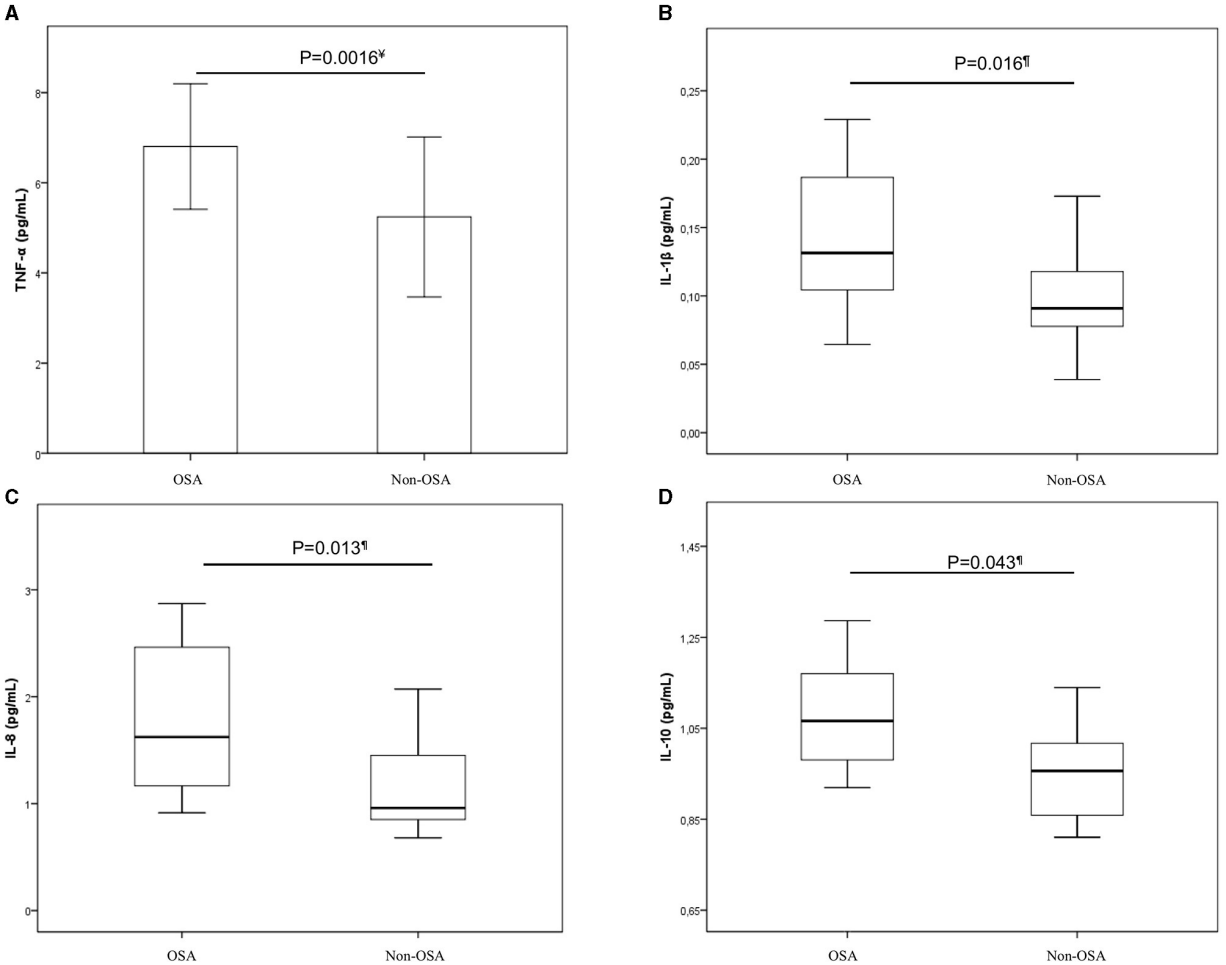
Comparison of cytokines levels in OSA and non-OSA pregnant women. Comparison of cytokines levels in OSA and non-OSA pregnant women. Statistical tests: *t*-Student^¥^; *U* Mann- Whitney^¶^. **(A)** TNF-α^¥^; **(B)** IL-1β^¶^; **(C)** IL-8^¶^; **(D)** IL-10^¶^. The bar chart represents mean and standard deviation (TNF-α) and boxplots represent medians and interquartile ranges (IL-1β, IL-8, and IL-10). IL, interleukin; OSA, Obstructive sleep apnea; TNF-α, Tumor necrosis factor alpha.

Pregnant women with OSA had higher levels of TNF-α, IL-1β, IL-8, and IL-10 compared to pregnant women without OSA. However, IL-6 was not significantly different (Table 3, Figure 1). Considering that the comparative study based on AHI revealed significant differences in TST, corrected comparisons were made applying a general linear model, and the existing differences in TNF-α, and IL-8 retained its statistical significance (6.91 ± 0.54 vs. 5.19 ± 0.37 pg/mL, *p* = 0.016; 1.97 ± 0.24 vs. 1.17 ± 0.16 pg/mL, *p* = 0.012, respectively), but IL-1β and IL-10 no longer reached statistical significance. Additionally, the study groups were homogeneous in their anthropometric characteristics, corrected comparisons were made adjusted for BMI before pregnancy and gestational age at plasma sampling, and BMI and gestational age at plasma sampling as covariate factors, respectively, and the existing differences in TNF-α, and IL-8 remained its statistical significance (Supplementary Tables 1, 2).

**Table 3:** Inflammatory profiles in pregnant women with and without OSA.

**Variables**	**OSA** **(***n*** = 11)**	**Non-OSA** **(***n*** = 22)**	***P*** **-value**
TNF-α (pg/mL)	6.80 ± 1.39	5.24 ± 1.77	**0.016^¥^**
IL-1β (pg/mL)	0.13 (0.10–0.18)	0.09 (0.07–0.12)	**0.013^¶^**
IL-6 (pg/mL)	0.97 (0.59–1.36)	0.66 (0.50–0.74)	0.067^¶^
IL-8 (pg/mL)	1.62 (1.11–2.57)	0.96 (0.85–1.53)	**0.016^¶^**
IL-10 (pg/mL)	1.07 (0.97–1.20)	0.96 (0.86–1.02)	**0.043^¶^**

*Continuous variables in mean ± sd if they follow normal distribution or in median (IQR) if they follow a non-parametric distribution. Statistical tests: t-Student^¥^; U Mann- Whitney^¶^; Chi-square^∞^. TNF-α, tumor necrosis factor alpha; IL, interleukin; pg, picograms; mL, milliliter. Values in bold indicate significant difference*.

### Correlations Between the Inflammatory Profile and Sleep Characteristics

**Figure 2:**
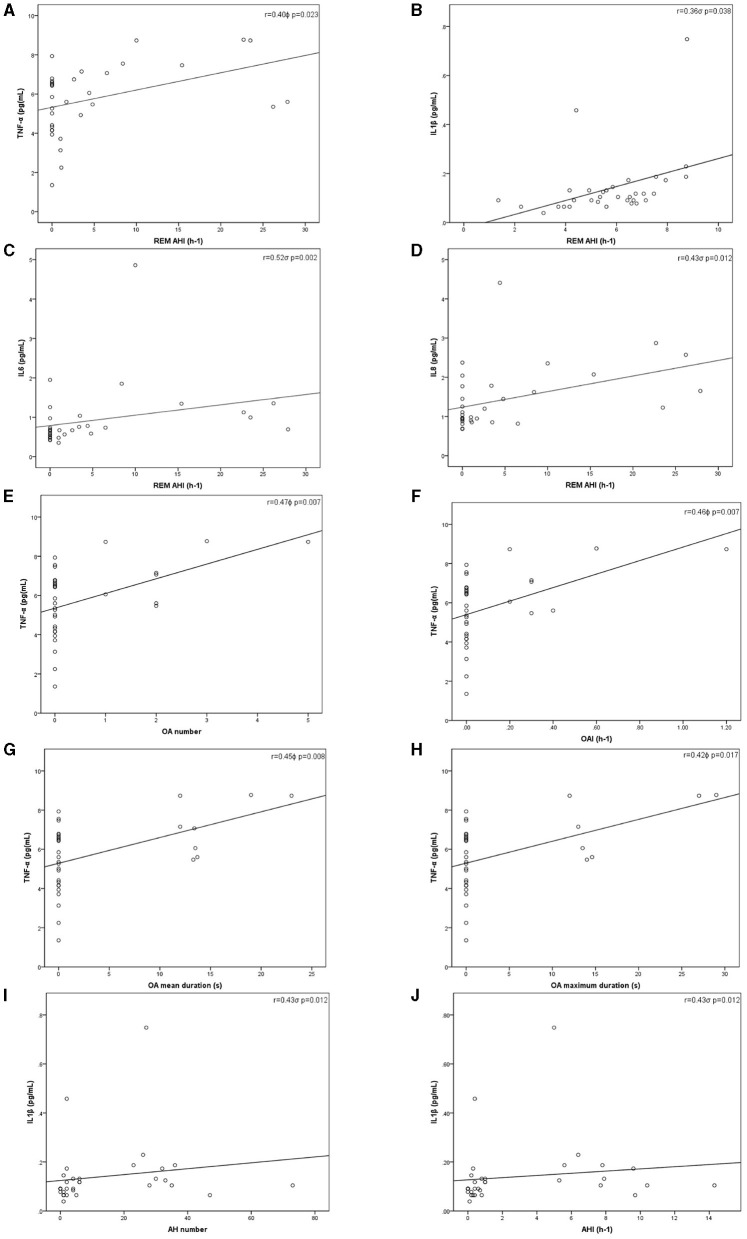
Inflammatory cytokines levels and sleep study parameters correlations. Pearson^ϕ^ (TNF-α) and Rho de Spearman^σ^ (IL-1β, IL-6, IL-8, and IL-10) correlations. **(A)** Correlation between TNF-α and REM AHI^ϕ^; **(B)** Correlation between IL-1β and REM AHI^σ^; **(C)** Correlation between IL-6 and REM AHI^σ^; **(D)** Correlation between IL-8 and REM AHI^σ^; **(E)** Correlation between TNF-α and the total number of OA^ϕ^; **(F)** Correlation between TNF-α and OAI^ϕ^; **(G)** Correlation between TNF-α and mean duration of OA^ϕ^; **(H)** Correlation between TNF-α and maximum duration of OA^ϕ^; **(I)** Correlation between IL-1β and the total number of AH^σ^; **(J)** Correlation between IL-1β and AHI^σ^. AHI, Apneas-hypopneas index; IL, Interleukin; OA, Obstructive apneas; OAI, Obstructive apneas index; *p, p*-value; *r*, Correlation coefficient; REM, Rapid eye movement; TNF-α, Tumor necrosis factor alpha.

Significant positive correlations were identified between AHI during REM and TNF-α (*r* = 0.40, *p* = 0.023), IL-6 (*r* = 0.52, *p* = 0.002), IL-8 (*r* = 0.43, *p* = 0.012), and IL-1β (*r* = 0.36, *p* = 0.038) concentrations. Significant positive relationships were also found between index, total number, maximum, and mean duration of obstructive apneas and TNF-α (*r* = 0.46, *p* = 0.007; *r* = 0.47, *p* = 0.007; *r* = 0.42, *p* = 0.017, *r* = 0.45, *p* = 0.008, respectively). Total number of apneas-hypopneas, and AHI were also related to IL-1β values (*r* = 0.43, *p* = 0.012; *r* = 0.43, *p* = 0.012, respectively) (Figure 2).

We found a mild correlation between CT90% and IL-8 (*r* = 0.37, *p* = 0.036). No more relationships between nocturnal hypoxemia markers and plasma cytokines were found. Neither recorded TST nor arousal index were significantly associated with cytokine values.

### Correlations Between the Inflammatory Profile and Neonatal Characteristics

**Figure 3:**
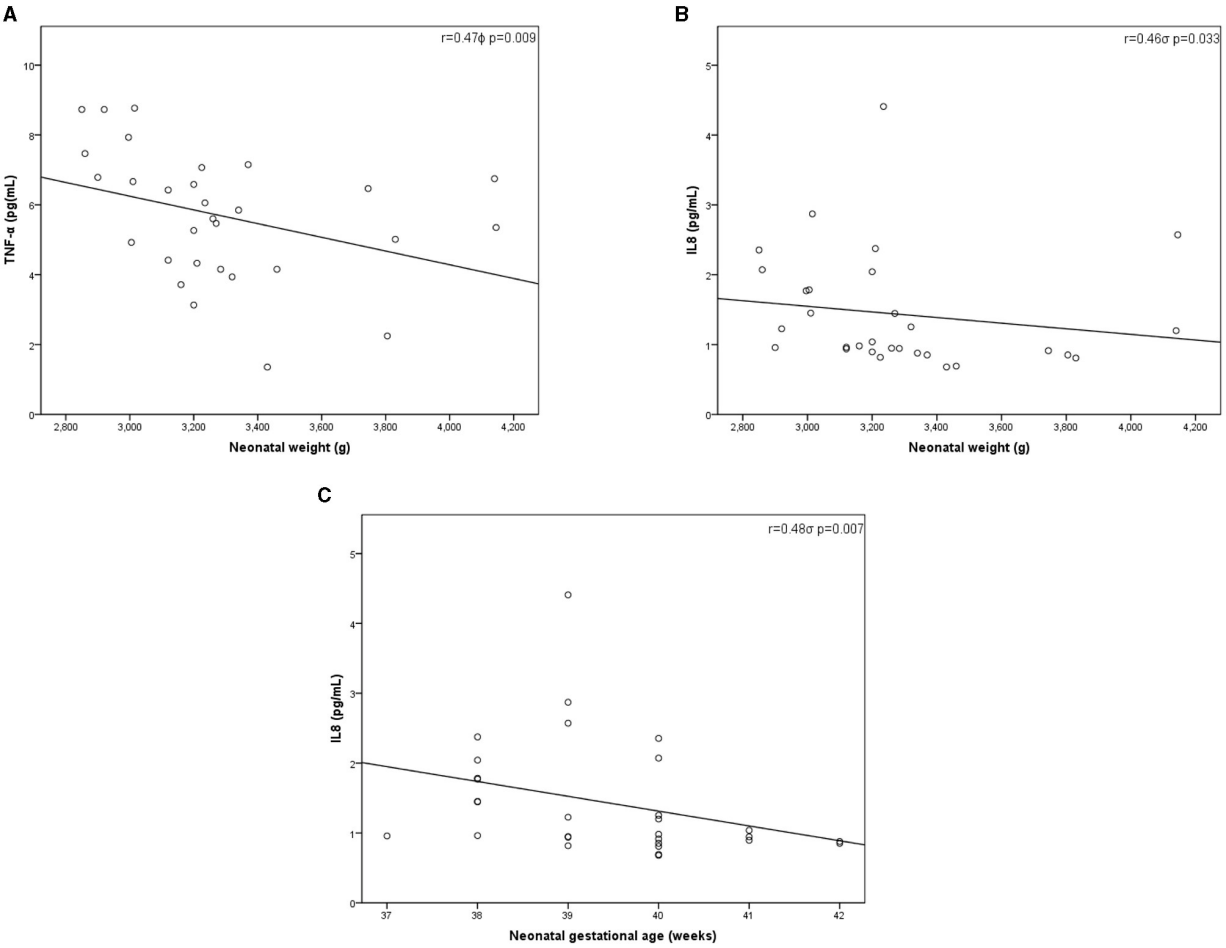
Inflammatory cytokines levels and neonatal characteristics correlations. Pearson^ϕ^ (TNF-α) and Rho de Spearman^σ^ (IL-8) correlations. **(A)** Correlation between TNF-α and neonatal weight^ϕ^; **(B)** Correlation between IL-8 and neonatal weight^σ^; **(C)** Correlation between IL-8 and neonatal gestational age^σ^. IL, Interleukin; *p, p*-value; *r*, Correlation coefficient; TNF-α, Tumor necrosis factor alpha.

Neonatal outcomes are presented in Table 4. None of them had preterm birth (median neonatal gestational age 39.5 (IQR 38–40) weeks), fetal trauma; needed oxygen therapy, phototherapy or were admitted to intensive care units. We did not find significant differences in the neonatal characteristics based on the presence of OSA in pregnant women as shown in Table 4. However, when examining the effects of TNF-α, and IL-8 on neonatal birth weight, we found significant associations with higher cytokine levels and lower birth weight (Figure 3). A significant inverse significant correlation between IL-8 and neonatal gestational age (*r* = –0.48, *p* = 0.007) was also found.

**Table 4:** Neonatal characteristics in OSA and non-OSA groups.

**Variables**		**Total**	**OSA**	**Non-OSA**	***P*** **-value**
Birth weight (Kg)		3.21 (3.01–3.39)	3.25 (2.94–3.62)	3.21 (3.09–3.39)	0.963^¶^
Birth weight (percentile)		51.2 ± 23	51.9 ± 29.8	50.9 ± 20.9	0.870^¥^
Birth height (cm)		49.2 ± 1.7	48.75 ± 2.1	49.3 ± 1.7	0.600^¥^
Head circumference (cm)		35 (34–36)	34 (33–34)	35 (34–36)	0.215^¶^
Umbilical cord pH		7.30 ± 0.07	7.30 ± 0.05	7.29 ± 0.07	0.739^¥^
Apgar 1st min		9 (9–9)	9 (8–9)	9 (9–9)	0.233^¶^
Apgar 5th min		10 (10–10)	10 (9–10)	10 (10–10)	0.590^¶^
Gender *n* (%)	M	14 (46.7)	4 (50)	10 (45.5)	1.000^∞^
	F	16 (53.3)	4 (50)	12 (54.5)	
Neonatal gestational age (weeks)		39.5 (38–40)	39 (39–39.8)	40 (38–40.3)	0.383^¶^

*Continuous variables in mean ± sd if they follow normal distribution or in median (IQR) if they follow a non-parametric distribution. For categorical variables n (%). Statistical tests: t-Student^¥^; U Mann- Whitney^¶^; Chi-square^∞^. M, male; F, female; Kg, kilogram; cm, centimeter; min, minutes*.

## Discussion

Emerging evidence suggests that OSA prevalence increases (6, 7) during pregnancy, and it is associated with adverse pregnancy-related outcomes, such as gestational hypertension, GDM, pre-eclampsia, preterm birth and low birth weight (8, 11, 12, 14, 29). The mechanisms responsible for these associations have not yet been fully elucidated. Among others, it has been proposed that systemic inflammation may play a role as an intermediate potential mechanism (30, 31). However, until now, there were no studies on non-complicated pregnant women with OSA that had specifically investigated the effects on inflammation.

### Principal Findings

Our study provides three main findings of interest. First, women with OSA during pregnancy had significant higher levels of TNF-α, IL-1β, IL-8, and IL-10 compared to women without OSA. Second, there was a significant correlation between systemic inflammation and respiratory obstructive sleep events, especially during REM sleep. Third, systemic inflammation during the third trimester was inversely correlated with neonatal birth weight and newborn gestational age.

### Sleep Symptoms in OSA and Non-OSA Groups

There were no differences in daytime sleepiness and in OSA symptoms between pregnant women with OSA and control subjects. ESS shows low sensitivity and specificity, and it is poorly predictive of OSA during pregnancy (32, 33). Reasons that could explain its weakness to identify OSA included the fact that it was designed for a non-pregnant population, and that sleepiness is a common complaint during pregnancy that may be multifactorial and not specific for OSA. Additionally, pregnant women with OSA may not suffer from daytime sleepiness. Similarly, we did not find significant differences in other OSA symptoms, although there was a clear trend in some of them (frequent gasping awakenings, morning headaches) (Table 2). Our study aim was to compare the levels of inflammatory cytokines in pregnant women with and without OSA, and the sample size of the present study may be not sufficiently powered to find differences in the sleep symptoms.

### Inflammatory Profile and OSA

Many studies have shown that OSA in non-pregnant populations increases the concentration of some pro-inflammatory cytokines such as TNF-α, IL-1β, IL-6, and IL-8, among others (34–37). However, there are contradictory results that may be due to significant influence of age, weight, gene polymorphisms, or lifestyle. Similarly, there are conflicting data in IL-10 levels. Some studies found that they are lower in OSA (38) while others conclude that there were no significant differences between OSA and healthy patients (34). The third trimester of pregnancy is characterized by a low grade inflammation, which is necessary for the correct evolution throughout pregnancy (16). Specifically, both the levels of pro-inflammatory cytokines such as IL-1β, IL-6, and IL-8 (15–17) as well as anti-inflammatories such as IL-10 (39) increase during healthy pregnancy. However, there are many discrepancies in the previous studies and even in the quantified values, which change within the trimesters of gestation, comorbidities, obesity, percentage of visceral adipose tissue, and treatments. Besides, the variation coefficients of the different techniques used, most of them by means of enzyme immunoassays in solid phase, as they are semi-automated and with high inaccuracies, can originate different measurements among the reported studies. It is important to point out that there is not an international standardization in the techniques to report harmonized results either. However, some other groups reported similar cytokine concentrations to our study values (19, 39–46). In the present study there were no differences in age or BMI, which set aside important confounders, such as maternal age and obesity when evaluating inflammation. We found that OSA pregnant women presented higher values of TNF-α, IL-1β, IL-8, and IL-10 cytokines compared to non-OSA ones. Moreover, there were significant and positive relationships between some inflammatory markers and the number of obstructive respiratory events per hour during sleep. As far as we know, this is the first description on the influence of repetitive nocturnal obstructive apneas events in systemic inflammation in non-complicated pregnant women.

Interestingly, we found significant positive correlations between AHI during REM and TNF-α, IL-6, IL-8, and IL-1β. REM sleep accounts for around 25% of total sleep time in healthy adults. OSA isolated to REM sleep in general population has been independently associated with prevalent and incident hypertension, as well as with insulin resistance (47, 48). OSA events during REM sleep are usually longer than those during non-REM sleep and are often followed by more pronounced hypoxemia, consequently they could be more harmful. In addition, REM sleep is associated with intensified sympathetic activation, which may further upgrade negative physiologic responses. OSA in REM prevalence ranges between 10 and 36%, being more frequent in women, younger individuals and those with mild to moderate OSA (49). We speculate that REM-OSA during pregnancy may lead to upregulation of the inflammatory response, which might intensify the risk of worse maternal-fetal outcomes. Nonetheless, future studies are required to further explore the role of REM-OSA and these inflammatory markers during pregnancy as well as their influence in hypothetical human gestation complications.

We found a mild correlation between CT90% and IL-8. No more significant relationships between nocturnal hypoxemia indexes and plasma cytokines were found. Consequently, pregnancy inflammation response to OSA may be more susceptible to respiratory obstructive sleep events, especially during REM sleep rather than to during nocturnal hypoxemia. However, studies to a greater extent are needed to better determine the influence of nocturnal hypoxemia on systemic inflammation, given that women of the present study presented very mild impact of OSA on nocturnal hypoxemia indexes, limiting any other further analysis (Table 2).

TST (objectively measured by PSG) was significantly shorter in the OSA group. Nevertheless, there was no clear external cause for worsened sleep quality in the OSA group, and significant differences in subjective self-reported sleep time were not found either. Corrected comparisons were made applying a general linear model. TNF-α, and IL-8 remained higher in the OSA group compared to women with AH < 5 h^−1^, but IL-1β and IL-10 no longer reached statistical significance. However, neither TST nor arousal index were significantly associated with cytokine values. Other studies found that women with GDM or gestational hypertension had markedly lower TST, and a higher AHI (with higher proportion of OSA) than women with uncomplicated pregnancies (50, 51). Similarly, a study of 234 pregnant women showed a trend toward decreased TST as the severity of AHI categories increased (52). We hypothesize that the short TST of the OSA women may have been a consequence of OSA-induced sleep disruption, and one of the intermediate mechanisms that may lead OSA to increase inflammation during pregnancy.

### Inflammatory Profile and Pregnancy and Neonatal Outcomes

Some inflammatory cytokines, such as IL-1β, IL-6, IL-8, and TNF-α, have been implicated in the pathogenesis of pregnancy complications such as pre-eclampsia and GDM (18–21). Similarly to us, Bublitz et al. showed higher levels of IL-6, IL-8, and TNF-α in a small group of women with GDM with OSA compared to women without OSA (53). Nevertheless, the findings of the latter study should be interpreted cautiously since, despite there were no differences in BMI, they included only 4 women with OSA with mean BMI of 36.4. Moreover, OSA status was diagnosed at home with a type III portable device, which has not been validated in pregnant women.

IL-10 is an immunosuppressive cytokine that plays an important role in controlling the equilibrium of pro- and anti-inflammatory response, essential for pregnancy maintenance and development, as well as for placental growth and remodeling for a successful pregnancy (40). Our results showed higher levels of IL-10 in pregnant women with OSA compared to control subjects. Although there are conflicting studies, in the same way, many authors found that complicated pregnancies presented higher serum levels of IL-10 than healthy women (21).

In the present study we included mild OSA women (AHI median 7.8 h^−1^), which, however represent the typical range of OSA during pregnancy (8). Few studies have investigated the consequences of mild maternal OSA. Recent studies, in which OSA cases were mostly mild, found that even modest elevations of AHI were associated with an increased risk of hypertensive disorders and GDM (8, 50). Nonetheless, other studies showed contradictory results on the effects of mild OSA in a composite of adverse pregnancy outcomes (54), or in the GDM/pre-eclampsia risk (55). Birth weight is a critical fetal outcome, and a surrogate indicator of the intrauterine environment. The relationship between objective measures of mild OSA and birth weight has been hardly studied with mixed results and remains currently unclear. Some studies suggest that maternal OSA in pregnancy (even in a mild form) is associated with reduced fetal growth in late pregnancy (14), and with an increased risk of delivering a small-for-gestational-age infant (52), while other studies showed contrasting findings (56–58). Such inconsistencies may relate to differences in sample characteristics with heterogeneous populations, with inadequate control on confounders, such as pre-gestational BMI and pregnancy complications, and methodological differences in terms of sleep assessment procedures, as well as timing of sleep evaluation. As pregnancy is associated with mostly mild OSA, further study is warranted in these patients to better understand the mechanisms and consequences of OSA in this unique population.

Our study supports previous findings that showed an association of maternal cytokines (IL-8 and TNF-α) with perinatal complications (20, 22, 59). We showed a moderate correlation with lower neonatal birth weight and maternal IL-8 and TNF-α l levels. In the same way, maternal TNF-α concentration was inversely associated with birth weight in normal weight women (60). Human and animal studies also found that higher maternal plasma TNF-α concentration was associated with smaller birth weights (22, 61–63). However, there are conflicting results, as other studies did not find correlations with either birth weight or fetal growth restriction (64, 65). There is a heterogeneous inflammatory profile which may be related to the timing of pregnancy in when the samples were collected, the complexity in interpreting dynamic cytokine profiles, differences in assay methodologies, gene polymorphism or comorbidities, as well as diet and lifestyle among others (66). Our study aim was to compare the levels of inflammatory cytokines in pregnant women with and without OSA. Thus, the sample size of the present study may be not sufficiently powered to find differences in maternal and perinatal outcomes. Likewise, the median observed birth weight was 3,210 g, and none of the subjects met the criteria for low birth weight, which could limit further analysis to study. While we did not find significant differences in perinatal outcomes between OSA and non-OSA groups, we did find correlates of OSA and pro-inflammatory cytokines. It is plausible that maternal systemic inflammation related to OSA, although very mild, may disturb growth pattern of the offspring. It is however unlikely that a single mechanistic pathway could modify birth weight. Increments in oxidative stress, and in sympathetic activation, modifications in the angiogenic balance, and endothelial dysfunction may all play a role. Notwithstanding such considerations, the impact of OSA during pregnancy on fetal outcomes and whether OSA-related inflammation could be one intermediate mechanism have yet to be determined with further studies presenting a larger sample size.

### Strengths and Limitations

The present study has several strengths, such as novelty, a multi-center approach, prospective enrolment, careful selection of patients and controls in which comorbidities were carefully excluded, who were homogeneous in age and body mass index (BMI), and intensive sleep and clinical phenotyping, including the performance of attended PSG. Additionally, the laboratory researchers were blinded to maternal status. Yet, as in any study, there are some potential limitations that deserve to be commented on. First, the sample size was relatively small. This was due to the difficulty of including pregnant women with newly diagnosed OSA, but otherwise healthy (prevalence is around 10%) (8). Despite this fact, the number of individuals included was sufficiently powered to observe significant differences. Second, since our study included mainly Caucasian women, our results may not be directly applicable to other ethnic groups. Third, since episodes of flow limitation <30% associated with arousal were not scored, we cannot ascertain whether our results would still be similar.

## Conclusions

To conclude, we have found that pregnant women with OSA without comorbidities had higher systemic inflammation than healthy pregnant women without OSA, which was related to respiratory obstructive sleep events, especially during REM. In addition, the existence of systemic inflammation in pregnancy was inversely correlated with the newborn weight and age. Future studies are needed to further explore the hypothesis that increments in OSA-related maternal systemic inflammation could be an intermediate mechanism linking OSA during pregnancy and adverse maternal and perinatal outcomes, such as GDM, pre-eclampsia, or fetal birthweight (20). Furthermore, the influence and possible correlation of maternal inflammation on the fetal inflammation markers still needs to be tested. Lastly, additional data regarding the progression of the inflammatory profile of pregnant women after delivery, as well as the potential influence if the OSA condition during pregnancy is corrected, are needed to better assess the risk of OSA-related systemic inflammation during pregnancy.

## Data Availability Statement

Data cannot be made publicly available for ethical reasons as study participants did not give consent for public data sharing: due to the ethical restrictions related to the consent of the participants of the public deposition of the data, as well as legal limitations of the Spanish legislation on data protection, it is not possible to have the permissions to publically access the database. However, other researchers who meet the criteria for access to confidential data, may request to gain access to the minimal data set underlying the results under request at the Ethics Committee (ceic_ib@caib.es), which will evaluate the data request proposal within the meaning of Medical Research involving Human Subjects.

## Ethics Statement

The studies involving human participants were reviewed and approved by Comité de Ética de La Investigación de Las Islas Baleares (CEI-IB). The patients/participants provided their written informed consent to participate in this study.

## Author Contributions

AA-F, JS, and FG-R conceived and designed the study, contributed to data interpretation, and writing the manuscript. MCe, AA, AS, MCo, PR, CR, AH, MG, CP, AG, MB, MD, AB, DM-G, AI, JD, JP, JC, and JM contributed to literature search, to recruit patients, to data analysis and interpretation, and writing the manuscript. All authors contributed to the article and approved the submitted version.

## Funding

This research was partially supported by grants from SEPAR-2010-820, Ministerio de Economía y Competitividad (PI10/00495) and Ministerio de Ciencia, Innovación y Universidades (PI19/00875).

## Conflict of Interest

The authors declare that the research was conducted in the absence of any commercial or financial relationships that could be construed as a potential conflict of interest.

## Publisher's Note

All claims expressed in this article are solely those of the authors and do not necessarily represent those of their affiliated organizations, or those of the publisher, the editors and the reviewers. Any product that may be evaluated in this article, or claim that may be made by its manufacturer, is not guaranteed or endorsed by the publisher.

## Abbreviations

AHI: Apnea-hypopnea index
ALT: Alanine aminotransferase
AST: Aspartate aminotransferase
BMI: Body mass index
BP: Blood pressure
CT90%: Sleep time with oxygen saturation <90%
GDM: Gestational diabetes mellitus
GGT: Gamma-glutamyl transpeptidase
HDL: High-density lipoprotein
HOMA-IR: Homeostatic model assessment of insulin resistance
IH: Intermittent hypoxia
IL: Interleukin
IQR: Interquartile range
LDL: Low-density lipoprotein
OAI: Obstructive apnea index
OSA: Obstructive sleep apnea
PSG: Polysomnography
REM: Rapid eye movement
Sd: Standard deviation
SpO_2_: Oxygen saturation
TG: Triglycerides
TNF-α: Tumor necrosis factor alpha
TST: Total sleep time.
