# Supplementary material for: Influence of Obstructive Sleep Apnea on Systemic Inflammation in Pregnancy
Source: Front Med (Lausanne). 2021 Nov 2;8:674997. doi: 10.3389/fmed.2021.674997 (PMC8593073; doi:10.3389/fmed.2021.674997)
Supplement: Supplementary file 1 [file Data_Sheet_1.docx]

**Table S1. Inflammatory profiles in pregnant women with and without OSA adjusted for BMI before pregnancy and gestational age at plasma sampling**

| **Variables** | **OSA (n=11)** | **Non-OSA (n=22)** | **p.value** |
| --- | --- | --- | --- |
| **TNF-α (pg/mL)** | 6.64±0.47 | 5.23±0.31 | **0.019** |
| **IL-1β (pg/mL)** | 0.18±0.04 | 0.12±0.03 | 0.247 |
| **IL-6 (pg/mL)** | 0.93±0.13 | 0.74±0.09 | 0.235 |
| **IL-8 (pg/mL)** | 1.85±0.24 | 1.20±0.16 | **0.034** |
| **IL-10 (pg/mL)** | 1.04±0.1 | 0.98±0.07 | 0.615 |

Variables in estimated marginal means ± se. Abbreviations: TNF-α, tumor necrosis factor alpha; IL, interleukin; pg, picograms; mL, millilitre.

**Table S2. Inflammatory profiles in pregnant women with and without OSA adjusted for BMI and gestational age at plasma sampling**

| **Variables** | **OSA (n=11)** | **Non-OSA (n=22)** | **p.value** |
| --- | --- | --- | --- |
| **TNF-α (pg/mL)** | 6.64±0.49 | 5.23±0.32 | **0.024** |
| **IL-1β (pg/mL)** | 0.18±0.04 | 0.12±0.03 | 0.223 |
| **IL-6 (pg/mL)** | 0.92±0.13 | 0.74±0.09 | 0.254 |
| **IL-8 (pg/mL)** | 1.83±0.24 | 1.22±0.16 | **0.048** |
| **IL-10 (pg/mL)** | 1.03±0.1 | 0.98±0.07 | 0.649 |

Variables in estimated marginal means ± se. Abbreviations: TNF-α, tumor necrosis factor alpha; IL, interleukin; pg, picograms; mL, millilitre.
